# New Treatment Options for Pancreatic Neuroendocrine Tumors: A Narrative Review

**DOI:** 10.3390/cancers17233837

**Published:** 2025-11-29

**Authors:** Agnieszka Romanowicz, Marta Fudalej, Alicja Asendrych-Woźniak, Anna Badowska-Kozakiewicz, Paweł Nurzyński, Andrzej Deptała

**Affiliations:** 1 Department of Oncology, National Medical Institute of the Ministry of the Interior and Administration, 02-507 Warsaw, Poland; agnieszka.romanowicz@pimmswia.gov.pl (A.R.); marta.fudalej@wum.edu.pl (M.F.);; 2 Department of Oncological Propaedeutics, Medical University of Warsaw, 01-445 Warsaw, Poland

**Keywords:** pancreatic neuroendocrine tumors, peptide receptor radionuclide therapy, targeted therapy, immunotherapy, narrative review

## Abstract

Pancreatic neuroendocrine neoplasms (PanNENs) are a diverse group of cancers with varying clinical presentations and prognoses due to differences in morphology and clinical stage. Most are non-functional tumors that express somatostatin receptors (SSTRs). Several treatment options have been established for patients with locally advanced or metastatic PanNETs, but the optimal choice of treatment approach and the sequence of available therapies are not yet clearly defined and are currently being studied in multiple ongoing clinical trials. Additionally, new drugs are being researched for PanNET treatment, including immune checkpoint inhibitors, next-generation peptide receptor radionuclide therapy, and other targeted biological therapies. To improve treatment outcomes for patients with PanNETs, a multidisciplinary team should evaluate systemic treatment options. The aim of this article is to review currently available therapies and discuss new and emerging systemic treatment strategies for patients with advanced PanNETs.

## Simple Summary

Pancreatic neuroendocrine neoplasms (PanNENs) are a rare group of tumors with a prognosis related to their proliferative activity and clinical stage at diagnosis. Based on morphological differentiation and grading, PanNENs are classified as well-differentiated neuroendocrine tumors (PanNETs) of low grade (G1), intermediate grade (G2), and high grade (G3), as well as poorly differentiated pancreatic neuroendocrine carcinomas (PanNECs). Surgical resection is the most effective and potentially curative treatment option for PanNETs. Unfortunately, neuroendocrine neoplasms (NENs) are most often diagnosed at advanced stages and are not suitable for surgical treatment. For these cases, systemic treatment options are available, including somatostatin analogs, molecularly targeted drugs, chemotherapy, and peptide receptor radionuclide therapy. Many ongoing clinical trials are exploring new agents, either alone or in combination, to expand treatment options. This article provides an overview of current treatment methods for PanNETs and future perspectives in this field.

## 1. Introduction

Neuroendocrine neoplasms (NENs) are a heterogeneous group of malignancies that arise from the secretory cells of the diffuse neuroendocrine system [1]. They most commonly occur in the digestive system, including the gastrointestinal tract (GI-NET) and pancreas (PanNET), which is the most common site for gastroentero-pancreatic neuroendocrine neoplasms (GEP-NENs) [2].

Although their rarity, the incidence of PanNENs has increased significantly over the past few decades and is estimated at 0.48 per 100,000 new cases per year [3]. In the region’s database, which is more similar to the Polish population—the German database (1976–2000)—there were 2821 recorded cases of neuroendocrine neoplasms, with a gender distribution of 45.8% male and 54.2% female [4]. PanNENs account for 4% of all NENs, and they represent 1–2% of all pancreatic neoplasms [5]. A global increase in the incidence of NENs is observed across most primary sites and affects both low- and high-grade NENs [6]. Among risk factors for sporadic PanNENs, comorbidities, first-degree family history of any cancer, smoking, and alcohol use were the most frequently explored [7]. Some of these have been identified as risk factors for sporadic non-functional pancreatic neuroendocrine tumors (NF-PanNETs), including a first-degree family history of any cancer, smoking, and diabetes, while heavy drinking was associated with functional PanNETs [8]. In a recent study, it was shown that the number of comorbidities among patients with gastroentero-pancreatic neuroendocrine tumors (GEP-NETs) and lung NETs was greater than for a similar group of people without a cancer diagnosis. Hypertension and diabetes mellitus were the most frequent [9]. Other diseases occurring in patients with a NET include hyperlipidemia, respiratory and renal failure, and secondary neoplasms of the gastrointestinal system [10]. Recent analyses investigating the possible link between cardiovascular disorders and PanNETs show no evidence of an association [11,12]. The presence of comorbidities should be carefully investigated in main medical decisions, especially among patients treated with inhibitors of serine/threonine-specific protein kinases, which can cause metabolic disorders [13].

Most often, PanNENs occur sporadically and are diagnosed at an older age (between the 40s and 60s) than in cases of PanNENs associated with inherited syndromes [14]. NENs, particularly well-differentiated, slow-growing tumors (NETs), can also occur in the context of hereditary syndromes, including multiple endocrine neoplasia type 1 (MEN1), von Hippel–Lindau syndrome (VHL), and neurofibromatosis 1 (NF-1), which is often linked to PanNETs [15]. The development of PanNETs is related to genetic mutations that are involved in critical processes such as cell growth regulation, DNA repair, and genome stability maintenance [16]. The most common of these are mutations in genes related to *MEN1*, death-domain-associated protein (*DAXX)*, α thalassemia/mental retardation syndrome X-linked (*ATRX)*, and the mammalian target of rapamycin (mTOR) pathway, leading to abnormalities in cell growth and differentiation that cause uncontrolled proliferation [17,18]. A deeper understanding of genetic alterations as well as epigenetic modifications in PanNETs may provide the basis for promising future research [19]. The most advanced PanNETs (60–90%) are non-functional tumors due to the absence of symptoms caused by the overproduction of specific hormones, and therefore, clinical symptoms may be depend on the tumor burden and the primary origin of the neoplasm [20,21,22]. The majority of patients (64%) with PanNETs have distant metastases, and the median survival in this group is 24 months [5]. The most common site of metastases is the liver, where they are present in at least 10% of patients at the time of diagnosis [23]. Surgical resection of localized disease is the most optimal and curative therapeutic option for patients with PanNETs and neuroendocrine carcinomas (NECs) and offers the best chance of cure [24], although it is also associated with significant morbidity. Therefore, the choice of this treatment option should be carefully considered [25]. Moreover, PanNETs are often diagnosed at an advanced stage due to their slow growth and long asymptomatic course, which is a limitation for radical surgical treatment [26]. Among palliative therapies used in patients with advanced or metastatic PanNETs, somatostatin analogs (SSAs) play an important role in initial treatment according to their good tolerability and proven efficacy for both controlling hormonal symptoms and inhibiting tumor growth, particularly in the case of slow-growing, non-functional G1 and G2 PanNETs (NF-PanNETs) with positive somatostatin receptor (SSTR) status [27,28].

Peptide receptor radionuclide therapy (PRRT) using α and β emitters, mTOR inhibitors, multikinase inhibitors, and chemotherapy are valuable options for the next stage of treatment [29]. The choice of the appropriate procedure depends on morphological differentiation, origin, grading of malignancy of the primary tumor, the Tumor, Node, and Metastasis (TNM) stage, functional activity, and SSTR expression [30]. As NETs are often diagnosed at an advanced stage, palliative treatment is the only option. However, its effectiveness and duration of response are limited among patients with PanNENs. Therefore, new therapeutic targets and innovative treatment strategies, including a personalized approach and an optimal therapeutic sequence, are urgently needed to improve the effectiveness of treatment for these tumors. A deeper understanding of the molecular biology of NETs provides an opportunity to explore new systemic targeted therapies for this condition [31].

The primary objective was to summarize currently available therapies and review emerging systemic treatment options for patients with an advanced PanNET. Secondary objectives were as follows:

− To highlight recent progress in diagnostic and treatment technologies, including advancements in digital technologies;
− To summarize the most recent advances in emerging systemic options, focusing on new molecularly targeted therapies and immunotherapeutic agents;
− To review novel approaches to PRRT and innovative PRRT technologies;
− To discuss clinical dilemmas involved in selecting the most appropriate treatment option.

Methodological details are provided in the Supplementary Materials.

## 2. Prognostic and Predictive Biomarkers in PanNENs

### 2.1. Typical Features and Molecular Biology

The prognosis of pancreatic neuroendocrine neoplasms depends not only on the clinical stage, including lymph node involvement and distant metastases, but also on tumor grading [5]. Based on grading (mitotic rate and Ki-67 index) and morphological differentiation, NENs are classified according to the 2022 World Health Organization (WHO) classification system for neuroendocrine neoplasms into well-differentiated neuroendocrine tumors (NETs) of low grade (G1, Ki-67 < 3%), intermediate grade (G2, Ki-67 < 3%), and high grade (G3, Ki-67 > 20%), as well as poorly differentiated NECs (G3, Ki-67 > 20%, often >70%), which are highly aggressive neoplasms with unfavorable outcomes [32]. Among genetic alterations linked to poor prognosis in NECs, mutations in TP53 and Rb1 that inactivate these genes are the most common [33]. A wide range of gene mutations have also been studied in PanNETs. The most common are germline mutations associated with MEN-1, DAXX/ATRX, VHL, and mTOR.

Platinum-based chemotherapy is recommended as the first-line treatment for advanced or metastatic pancreatic neuroendocrine carcinoma (PanNEC) [34], but for the heterogeneous group of PanNETs, there are many available strategies, and the goal is to introduce the most appropriate treatment in the optimal sequence for the right patient. Currently, the only clinically validated prognostic and predictive biomarker in PanNENs is SSTRs [35], whose expression determines the effectiveness of the targeted treatment with SSAs and is crucial for selecting patients for PRRT [36].

Most commonly, PanNETs arise sporadically, although nearly 20% of them, especially non-functional PanNETs, are associated with hereditary syndromes, including MEN1, VHL, and NF-1 [37].

Somatic mutations of the MEN-1 gene are responsible for tumor development in patients with MEN1 syndrome, but they have also been found in 44% of sporadic PanNEN cases. Another frequently mutated gene in PanNETs is ATRX/DAXX, which was identified in 43% of cases [38]. The loss of ATRX/DAXX complex activation has been shown to be related to chromosome instability and correlates with tumor aggressiveness, metastatic disease, and survival [39].

### 2.2. Molecular Targets and Signaling Pathways

A better understanding of the mechanisms that play key roles in tumor growth, progression, and response to therapies is crucial for selecting the appropriate treatment for each patient.

The mTOR pathway regulates many metabolic processes by integrating signals from various sources, including growth factors. It plays a role in cell differentiation and proliferation in normal cells, and its dysregulation is linked to the development of numerous diseases, including cancers [40]. The activity of the mTOR pathway is regulated by the phosphoinositide 3-kinase PI3K-Akt pathway, which is also frequently aberrant in various neoplasms [41]. Abnormal activation of the PI3K/Akt-mTOR pathway is responsible for the development of both familial and sporadic PanNETs [42]. Since the function of the mTOR pathway is often dysregulated in cancer cells, drugs targeting mTOR have become a valuable therapeutic option in these cancers [43].

Cyclin-dependent kinases (CDKs) constitute a family of kinases involved in the regulation of the cell cycle, serving as key regulators of various biological processes, including cell cycle progression, transcription, neuronal differentiation, and metabolism. Dysregulation of these kinases, resulting from gene amplification, protein overexpression, or mutations, leads to their hyperactivity and contributes to the uncontrolled proliferation characteristic of cancer cells. Among all the CDK/cyclin complexes, aberrations resulting in hyperactivity of the CDK4/cyclin D complex are observed in numerous human cancers, with the most prevalent being breast carcinoma, melanoma, osteosarcoma, liposarcoma, glioblastoma, and neuroblastoma [44]. The CDK4/6-cyclin D complex functions to phosphorylate the tumor suppressor Rb substrate, which regulates the cell cycle. Hyperactivity of the CDK4/6-cyclin D complex results in the loss of control in cell cycle regulation, leading to cell proliferation and cancer development. Consequently, CDK4/6 kinases constitute promising pharmacological targets [45]. The significant role of the CDK4/6 pathway in the development of NETs has been demonstrated in molecular studies [46].

Another signaling pathway involved in tumorigenesis is the Notch pathway. Notch signaling, which comprises transmembrane Notch receptors (Notch 1–4) activated by Notch ligands, participates in regulating the differentiation of stem cells and progenitor cells [47].

The role of Notch signaling in tumorigenesis varies across different solid tumors [48]. Activation of Notch promotes oncogenesis in ovarian cancer, colon cancer, lung adenocarcinoma, breast cancer, and pancreatic cancer, while in NENs, Notch suppresses the differentiation of neuroendocrine cells. Therefore, activation of this signaling pathway inhibits tumor growth in NENs [42]. Delta-like ligand 3 (DLL3) functions as an inhibitory ligand of the Notch pathway by interacting with Notch receptors (Notch1-4), promoting malignant tumor transformation. Its expression is minimal or absent in normal cells but is high in malignant NETs, which inhibits Notch1 and contributes to NEN development. Consequently, targeted therapies against DLL3 appear to be promising investigational options for NENs [49].

Regarding hypoxia markers, hypoxia inducible factors (HIF-1α and HIF2α) are among the most significant and promising targets. HIFs are transcription factors that play an essential role in regulating iron metabolism, erythropoiesis, and cellular growth. Consequently, dysregulation of these proteins can result in tumorigenesis and the progression of cancer [50]. The tumor suppressor protein VHL promotes the degradation of HIF-2 alpha under normal oxygen conditions. In hypoxic environments or when VHL is mutated, HIF induces increased proliferation and angiogenesis and contributes to cell growth [51]. The mutation of the VHL gene is observed in patients with VHL, who may develop various neoplasms, including PanNETs. PanNETs have been reported in 15–56% of patients with VHL [52].

The predictive biological biomarkers useful for selecting patients who may be appropriate for immunotherapy have been analyzed in many neoplasms where immunotherapy has proven effective. Among them, programmed cell death protein-1 ligand (PD-L1) expression, mismatch repair (MMR) status, and tumor mutational burden (TMB) were shown to be associated with response to immunotherapy [53]. In the systematic review of clinical trials conducted across various neoplasms treated with immunotherapy, high PD-L1 expression was identified as a predictive factor for response to immunotherapy treatment [54]. In non-small-cell lung cancer, elevated TMB was associated with a benefit from therapy with immune checkpoint inhibitors, independently of PD-L1 expression. Moreover, the co-occurrence of TMB and PD-L1 enhanced the response to immunotherapy [55]. Unfortunately, in PanNETs, the expression of PD-L1 was reported only in 7.4% cases [56]. The expression of PD-L1 correlated with tumor grade and was higher in G3 NETs and NECs [57].

## 3. Surgery and Locoregional Therapies

Radical resection of localized disease is the most optimal and curative therapeutic option for patients with GEP-NETs and NECs, offering the best chance for a cure. Surgical treatment may also benefit patients with distant metastases by providing symptom relief and improved survival through surgical reduction of tumor mass [58]. While radical resection of PanNETs has become the preferred surgical intervention, it is concurrently linked to considerable morbidity. Consequently, the selection of this treatment modality warrants meticulous deliberation [25]. For patients exhibiting non-functional tumors less than 10 mm in size, active surveillance constitutes a valuable option, but for NF-PanNETs larger than 20 mm, without distant metastases, surgical intervention is advised [5]. Unfortunately, PanNETs are frequently diagnosed at an advanced stage due to their slow growth and extended asymptomatic course, which limits the option of radical resection [26]. The most common site for distant metastases is the liver, observed in 50% of patients at the time of diagnosis. Furthermore, in 12–15% of cases, liver metastases develop subsequent to the curative excision of the primary lesion [23]. Palliative techniques, such as biliary bypass surgery, gastrojejunostomy, or debulking surgery, are performed in patients with advanced, metastatic, or unresectable PanNETs to enhance the quality of life [59]. Currently available locoregional therapies encompass liver-directed transarterial embolization (TAE), transarterial chemoembolization (TACE), and selective internal radiation therapy (SIRT) [60]. Some ablative techniques such as radiofrequency ablation (RFA), microwave ablation (MWA), and ethanol ablation (EA) can also be considered when a patient is not a candidate for surgery [61], and liver transplantation may be a valuable option for a strictly selected group of patients who have had the primary lesion removed and where the liver is the only site of metastases [62].

## 4. Chemotherapy

Systemic chemotherapy is a validated and approved treatment option for advanced PanNENs, especially in patients with quickly progressing metastases or significant symptoms caused by tumor burden [63]. The use of alternative systemic therapies, including somatostatin analogs and targeted agents like everolimus or sunitinib, has decreased the role of chemotherapy in managing low-grade (G1–G2) NETs. However, platinum-based cytotoxic therapy still remains the recommended first-line treatment for advanced NEC [64]. Alkylating agents, anthracyclines, pyrimidine analogs, and platinum-based therapies have been studied in the management of PanNENs through various single-agent and combination regimens. One of the first chemotherapeutic agents to show effectiveness in treating PanNETs was streptozocin [65]. Temozolomide-based therapies have replaced streptozocin-based regimens due to their lower toxicity and the convenience of oral administration [66]. The improved therapeutic outcome observed with the combination of temozolomide and capecitabine may be due to its ability to decrease O6-methylguanine DNA methyltransferase (MGMT) levels in tumor cells, thereby boosting the alkylating effectiveness of temozolomide [67]. Patients treated with temozolomide for glioblastoma multiforme showing low MGMT expression and MGMT promoter methylation exhibit improved response and survival [68]. However, the predictive value of MGMT remains unconfirmed in NET patients, and assessing MGMT expression is not required in clinical practice before using an alkylating agent [27].

Evidence from a randomized phase II trial demonstrated that combining temozolomide with capecitabine is effective compared to temozolomide alone for treating PanNETs [69]. Based on these results, administering temozolomide and capecitabine may be considered as an initial treatment for patients with PanNET G1/G2 who are SSTR negative or for those with symptomatic disease due to tumor burden or extensive disease volume [70]. For patients with PanNETs experiencing disease progression during capecitabine and temozolomide (CAP-TEM) therapy, switching to an alternative cytostatic regimen such as FOLFOX may be a favorable option, supported by clinical trial data. Retrospective analyses involving patients with metastatic PanNETs have also evaluated subsequent FOLFOX therapy, showing its effectiveness in this heavily pretreated population [71].

## 5. Development of SSA

Somatostatin is a peptide hormone that primarily inhibits many endocrine systems and regulates cell growth and hormone secretion by binding to one of five receptors (SSTR1–5) located on the cell membrane. These receptors are naturally expressed in a wide range of tissues but are also overexpressed in up to 80% of GEP-NET cases [72]. Activation of these receptors by SSAs suppresses intracellular signaling pathways, leading to reduced secretion, angiogenesis, and a cytostatic effect on tumor growth [73]. Although long-acting SSAs, including octreotide LAR and lanreotide Autogel, were originally used to manage the endocrine symptoms of NETs, their antitumor effects have been explored in several prospective clinical trials [74]. Both agents exhibit similar binding profiles across SSTR subtypes, with a particular affinity for SSTR2 [75]. The randomized controlled trials PROMID and CLARINET confirmed the role of somatostatin analogs in inhibiting NET growth [76,77]. Both studies indicated an improvement in disease progression rates with SSA treatment [65]. Based on these findings, octreotide LAR or lanreotide Autogel are recommended for initial treatment of slow-growing, SSTR-positive, advanced G1 and G2 NF-PanNETs [27]. Data from the phase 2 CLARINET FORTE trial suggest that an escalated dose of lanreotide (every 14 days) may be effective for patients with progressive NETs after standard first-line somatostatin therapy [78]. While octreotide LAR and lanreotide Autogel act by specifically binding to SSTR2, pasireotide is an SSA targeting multiple receptors, with affinity for SSTR1, 2, 3, and 5 [79]. In preclinical studies, pasireotide demonstrated superior antitumor activity compared to octreotide [80]. The efficacy of pasireotide in controlling tumor growth has been further evaluated in phase II and III studies [81]. A phase III trial comparing pasireotide LAR (60 mg) with octreotide LAR (40 mg) for treating carcinoid symptoms resistant to first-generation somatostatin analogs was terminated early due to an interim analysis that did not show pasireotide’s superiority regarding the primary endpoint, which was symptomatic response. Although pasireotide LAR showed a trend toward higher tumor control rates at six months, this was not statistically significant [82]. The combination of pasireotide and everolimus tested in the COOPERATIVE-2 trial in patients with advanced, progressive, and well-differentiated PanNETs did not improve progression-free survival (PFS) compared to everolimus alone [83]. Therefore, pasireotide in combination with everolimus is not recommended as standard therapy for patients with NETs [84]. Pasireotide LAR may be considered for patients with carcinoid syndrome who have not achieved adequate control with standard treatments [30,82].

## 6. Innovative PRRT Strategies

PRRT has demonstrated to be a highly effective and well-tolerated treatment option for advanced, inoperable, or metastatic well-differentiated GEP-NETs [85]. As previously mentioned, most PanNETs, especially well-differentiated NETs, express SSTRs 2 and 5.

This characteristic facilitates targeted PRRT for NETs and serves as an indicator of its potential efficacy [86]. Numerous retrospective studies have demonstrated the effectiveness of yttrium- or lutetium-labeled SSAs in patients with NETs [84]. Data from the NETTER-1 study contributed to the Food and Drug Administration (FDA)’s approval in 2018 of 177Lu-DOTATATE for the treatment of advanced somatostatin receptor-positive NETs, including gastrointestinal and pancreatic tumors, despite the fact that the trial did not include patients with PanNETs [87]. The rationale for this recommendation was based on data from the phase I/II ERASMUS study, which involved a cohort of 133 PanNET patients out of more than 1200 study participants treated with Lutetium [88,89]. PRRT is currently recommended as a second- or third-line treatment for progressive, SSTR-positive metastatic or inoperable grade 1 or 2 NETs [90].

The efficacy and safety of radioligand therapy as first-line treatment were recently evaluated in NETTER-2, a randomized phase III trial comparing (177Lu-Dotatate) combined with 30 mg octreotide LAR to 60 mg octreotide LAR in patients with advanced G2 and G3 GEP-NETs. This study was the first randomized trial of PRRT in patients with G3 NETs. The median PFS (mPFS) was 22.8 months, with a significantly extended duration observed in the cohort receiving 177Lu-Dotatate, in contrast to 8.5 months within the control group. No significant differences were observed across any subgroups. Combination therapy demonstrated a 72% decrease in the risk of disease progression or mortality compared to the cohort receiving a single high dose of octreotide. The authors concluded that PRRT can presently be regarded as a potential first-line treatment for patients with somatostatin receptor-positive G3 NETs; however, the question of whether it should become the standard first-line therapy for all G3 NET patients remains unresolved [91].

Another interesting study is the phase III COMPETE trial (NCT03049189), which compares PRRT with 177Lu-edotreotide to everolimus as first-line treatment for patients with progressive GEP-NETs with SSTRs. This study showed that 177Lu-edotreotide achieved a longer median PFS (23.9 months) compared to everolimus (14.1 months) in pre-treated advanced G1-G2 SSTR-positive GEP-NETs [92].

The ongoing COMPOSE trial (NCT04919226) seeks to assess the effectiveness and safety of PRRT with 177Lu-Edotreotide compared to everolimus or chemotherapy in patients diagnosed with well-differentiated higher-grade 2 and grade 3 GEP-NETs (NCT04919226).

Although PRRT is accepted for advanced and metastatic NENs, its role as a preoperative treatment remains uncertain [93]; nevertheless, this therapy appears to be a promising approach as a neoadjuvant treatment, particularly in patients with advanced synchronous liver metastases [94]. Given PRRT’s potential to shrink tumors, it has been studied as a downstaging method that may enable complete resection and reduce surgical complications [95]. Current published data from a multicenter phase II trial (NEOLUPANET) show that Lutetium is a safe and effective neoadjuvant therapy for patients with resectable or potentially resectable NF-PanNETs at high risk of recurrence. After four cycles of Lutetium, eighteen out of thirty-one patients experienced a partial radiological response, and thirteen showed disease stabilization. Twenty-eight patients underwent surgery, with twenty-four achieving R0 resection and four R1 resection. Seven patients experienced grade 3 postoperative complications, but there were no postoperative deaths. If these findings are confirmed in randomized clinical trials, this approach could become a promising treatment option [96].

For many years, SSAs used in PRRT were radiolabeled with β-emitting particles such as 90Yttrium (90Y) or 177lutetium (177Lu), which have suboptimal radiobiological properties. Targeted alpha particle therapy (TAT), a new generation of radioisotopes, is expected to be more effective and safer compared to β-particles due to its limited soft-tissue penetration and very high linear energy transfer, which increases the likelihood of double-strand DNA breaks [97,98].

Alpha emitters currently used in clinical trials involving patients with NENs include Actinium-225 (225 Ac)-DOTATATE, Bismuth-213 (213 Bi)-DOTATOC, and Plumbum-212 (212 Pb)-DOTAMTATE [99]. In a prospective study of 225Ac-DOTATATE, TAT was tested in 32 patients with advanced SSTR-positive GEP-NETs who had progressed after previous systemic treatment, including 177Lu-DOTATATE, or whose disease was stable after 177Lu-DOTATATE therapy. At a median follow-up of 8 months, there was no disease progression or death. Among the 24 patients who were assessed for morphological response, 15 showed a partial response, and 9 showed disease stabilization [100]. Evidence indicated a partial response to treatment with the radiopharmaceuticals (225Ac)-DOTATATE and (213Bi)-DOTATOC with moderate renal toxicity and mild hematological toxicity [101].

An innovative approach involves using PRRT for early response evaluation during the first cycle of radioligand therapy (RLT) in patients who later experienced disease progression [102]. A meta-analysis by Kim YI et al. showed that salvage PRRT is an effective treatment with toxicities similar to those of initial PRRT, including blood and kidney toxicities [103,104]. Retreatment with RLT, which has not yet been approved as a standard treatment, should be considered by the dedicated tumor board as a valid option for patients who responded positively to initial RLT [105].

## 7. Advances in Targeted Therapies

Targeted therapies are known to play a significant role in treating PanNETs. Among these, mTOR inhibitors and anti-angiogenic agents have shown activity and are approved for treating patients with advanced PanNETs [65].

Among the most common mutations in sporadic PanNETs, detected in 14% of tumors, are mutations in genes related to the mTOR pathway [106,107]. Since mTOR signaling plays a key role in cell growth, metabolism, and proliferation, mutations in the mTOR gene cause dysregulation of the pathway. This dysregulation is linked to carcinogenesis as well as the development and progression of PanNETs [18].

Everolimus is an oral inhibitor of the mTOR serine/threonine kinase signaling pathway [108]. The RADIANT-3 registration trial compared everolimus with a placebo in patients afflicted with progressive, advanced PanNETs. Everolimus significantly extended PFS among these patients. The mPFS was 11.0 months in the everolimus group, compared with 4.6 months in the placebo group [109].

Everolimus is recommended for use in patients with progressive G1 and G2 NF-PanNETs, in accordance with the current guidelines established by the European Neuroendocrine Tumor Society (ENETS), the American Society of Clinical Oncology (ASCO), and the European Society of Medical Oncology (ESMO). [27,30,70].

Combination therapy targeting the mTOR pathway with SSAs has also been evaluated to enhance antitumor activity in patients with PanNETs. In PanNETs, overactivity of the PI3K-Akt-mTOR pathway is thought to cause tumor initiation and progression [110]. Since SSAs inhibit PI3K activity by binding to SSR2, their use in combination with everolimus is expected to delay the time to progression [111].

The phase III STARTER-NET study conducted in Japan evaluated the combination of everolimus and lanreotide in patients with GEP-NETs and unfavorable prognostic factors. This study was predicated on the outcomes of uncontrolled phase II trials, which demonstrated that the combination of SSAs with everolimus improves PFS or postpones the progression of the disease [112]. The interim analysis demonstrates a notable extension of PFS in the combination therapy cohort relative to the monotherapy with everolimus, implying that this treatment approach may establish the new standard first-line therapy for patients with well-differentiated grade 1/2 GEP-NETs exhibiting adverse prognostic indicators [113].

NETs are considered highly vascularized tumors. Neuroendocrine cells overexpress proangiogenic factors, such as vascular endothelial growth factor (VEGF), platelet-derived growth factors (PDGFs) α and β, and fibroblast growth factor (FGF) [114]. VEGF is a proangiogenic molecule that plays an important role in the angiogenic process leading to carcinogenesis. Moreover, it promotes tumor growth and metastasis in solid neoplasm. Excessive activation of PDGF receptor beta (PDGFRβ) signaling induces autocrine stimulation of tumor cell growth and tumor angiogenesis [115].

Multikinase inhibitors that target proangiogenic factors are currently in phase 2/3 clinical trials.

Sunitinib malate, an oral small-molecule multitargeted tyrosine kinase inhibitor (TKI), targets vascular endothelial growth factor receptor (VEGFR), PDGFRs α and β, and the stem cell factor receptor (c-kit), providing strong anti-angiogenic and antitumor effects [116]. In a phase 2 clinical trial, sunitinib has shown activity in advanced PanNETs [117]. In a phase 3 study, sunitinib was compared with a placebo and demonstrated improved PFS. A statistically significant difference in the objective response rate (ORR) was also observed, with 9.3% in the sunitinib group compared to 0% in the placebo group [118]. Based on the findings of this study, the FDA authorized the use of sunitinib in 2010 for the management of progressive, well-differentiated PanNETs in patients with inoperable, locally advanced, or metastatic disease [119].

The SUNEVO study examined whether hypoxia caused by sunitinib could activate the pro-drug evofosfamide, turning it into a cytotoxic agent that kills cancer cells. This combination demonstrated moderate activity but had an unfavorable toxicity profile [120].

In addition to the approved multikinase inhibitor sunitinib, a new TKI targeting proangiogenic factors has shown promising clinical activity in PanNETs.

Cabozantinib is a tyrosine kinase receptor inhibitor that targets VEGFR2 and mesenchymal–epithelial transition (c-MET), thereby decreasing resistance to VEGFR inhibitors through the c-MET pathway [121]. A double-blind, phase III CABINET clinical trial assessed the effectiveness of cabozantinib in patients with progressive, previously treated (including therapy with Lu-177 DOTATATE or targeted agents such as everolimus and sunitinib) extrapancreatic neuroendocrine tumors (ePanNETs) or PanNETs. The results demonstrated a significant improvement in PFS compared to the placebo. The mPFS for patients with a PanNET was 13.8 months, compared with 4.4 months in the placebo group. No significant difference in overall survival (OS) was observed between treatment groups; however, OS data were not yet complete at the time of analysis. Crossover was permitted, and the rate of subsequent anticancer therapies was high [122]. The most frequently reported grade 3 or higher adverse events linked to cabozantinib encompassed hypertension, fatigue, diarrhea, and thromboembolic events [123].

The CABINET study presents cabozantinib as a valuable treatment option for NETs [124]. Based on the results from this study, on 26 March 2025, cabozantinib was approved by the FDA for patients with previously treated, advanced well-differentiated PanNETs and ePanNETs [59].

Currently, clinical trials are studying combinations of cabozantinib with lanreotide or temozolomide [NCT04427787; NCT04893785].

Surufatinib (HMPL-012), formerly known as sulfatinib, is an oral, small-molecule inhibitor of VEGFR1-3 tyrosine kinase that also targets fibroblast growth factor receptor 1 (FGFR1) and colony-stimulating factor 1 receptor (CSF-1R). This combined action inhibits tumor angiogenesis and modulates the immune microenvironment by interacting with tumor-associated macrophages [125].

Surufatinib was tested in a single-arm phase Ib/II trial involving advanced, well-differentiated, low-grade or intermittent-grade, inoperable or metastatic PanNETs and ePanNETs. The study demonstrated antitumor activity of surufatinib with manageable toxicity [126]. The drug was then examined in two randomized, double-blind, placebo-controlled phase III trials involving Chinese patients with well-differentiated, progressive, and advanced PanNETs (SANET-p) and ePanNETs (SANET-ep) [127,128].

In the SANET-p study, the primary endpoint of PFS was 10.9 months in the surufatinib group compared to 3.7 months in the placebo group. The most common grade 3/4 adverse events observed in the surufatinib-treated group included hypertension (38%), proteinuria (10%), and hypertriglyceridemia (7%). Treatment-related adverse events led to discontinuation of treatment in 18% of patients. The final analysis of both studies did not reveal a statistically significant improvement in OS in the surufatinib arm due to the crossover design of the study. The safety profile was consistent with the previous analysis, and no new safety findings were identified during the long-term follow-up of surufatinib [129]. Surufatinib was approved in China for the therapeutic management of locally advanced or metastatic, inoperable, progressive non-functional, and well-differentiated (G1 and G2) PanNETs and ePanNETs [130]. The pharmacokinetics, safety, and antitumor efficacy of surufatinib in United States patients with PanNETs and ePanNETs are similar to those observed in Chinese patients [131]. It therefore appears that surufatinib represents a new, valuable therapeutic option in subsequent lines of treatment for both PanNETs and ePanNETs [132].

Lenvatinib, which targets VEGFR1-3, FGFR-1-4 fibroblasts, RET, c-kit, and PDGFRα, is an oral small-molecule TKI [133]. In the phase II TALENT trial, lenvatinib was evaluated in patients with advanced grade 1/2 GEP-NETs after disease progression during treatment with mTOR inhibitors or TKI. For PanNETs, the centrally assessed primary endpoint ORR was 44.2%, the highest result reported for TKIs in this indication. The median PFS was 15.7 months, and the median duration of response was 19.9 months. Hypertension, fatigue, and diarrhea were the most common grade 3/4 adverse events. These encouraging results present lenvatinib as a potentially valuable option for treating PanNETs, particularly after the failure of previous targeted therapies [134].

Pazopanib, a multikinase inhibitor, targets VEGFR and PDGFR. It works by blocking signaling pathways, angiogenesis, and cell proliferation [135]. In a non-randomized, single-center phase II study, pazopanib was investigated in patients with metastatic GEP-NETs. In this study, pazopanib demonstrated efficacy in both PanNETs and GI-NETs, which was comparable to other targeted agents [136]. Pazopanib was also studied in a multicenter, open-label, phase II PAZONET trial in advanced, progressive, well-differentiated NET. The study revealed that twenty-five out of forty-four enrolled patients (59.5%) showed no disease progression after 6 months, and the median PFS was 9.5 months [137]. Future studies are needed to evaluate the efficacy and safety of pazopanib in the treatment of PanNETs.

Other proangiogenic molecules studied in NETs are HIFs, whose high expression levels in tissues correlate with liver metastases and significantly lower median OS in patients with PanNETs [138].

HIFs are transcription factors that play a pivotal role in regulating iron metabolism, erythropoiesis, and cellular growth. Consequently, dysregulation of these proteins can lead to tumorigenesis and cancer progression [50]. The tumor suppressor protein VHL promotes the degradation of HIF-2 alpha under normal oxygen conditions. In hypoxic environments or in the case of a VHL mutation, HIF induces the upregulation of angiogenesis and contributes to cell growth [51].

Belzutifan, a targeted inhibitor of the HIF signaling pathway that blocks HIF-2alpha [139], was initially studied in patients with renal cell carcinoma (RCC) and non-renal cell neoplasms, including PanNETs, associated with VHL disease [140]. In the primary analysis, treatment with belzutifan was associated with a 91% ORR among PanNETs with a VHL mutation [141]. Based on these results, the 2021 edition of the NCCN guidelines recommends belzutifan for treating patients with VHL-related tumors, including advanced PanNETs [142]. Currently, it is being evaluated in an ongoing phase II trial involving patients with advanced pheochromocytoma/paraganglioma (PPGL), PanNETs, VHL-associated tumors, advanced wild-type gastrointestinal stromal tumors (wt-GISTs), or advanced solid tumors with HIF-2α-related genetic alterations [NCT04924075].

## 8. Immunotherapeutic Agents

Immunotherapy has proven effective in treating many types of cancer (including lung cancer [143], melanoma [144], head and neck cancer [145], and RCC [146]), but its role in treating NETs remains unclears [147]. The use of this therapy in various endocrine tumors is limited to situations where conventional treatment options have failed [148].

Immune checkpoint inhibitors are a key part of immunotherapy strategies, but other approaches are also being explored, such as BiTE, chimeric antigen receptor (CAR) T cells, tumor-infiltrating lymphocytes, oncolytic viruses, and vaccines, aimed at patients with NENs [149].

Immune checkpoints, including cytotoxic T-lymphocyte-associated antigen 4 (CTLA)-4, the programmed cell death protein 1 (PD-1) receptor, and its ligands (PD-L1 and PD-L2), suppress T-lymphocyte activity, which can detect cancer antigens as foreign and destroy them [150]. Inhibiting these targets boosts immune system activity [151]; therefore, immunotherapy strategies should be investigated prospectively [152]. Among immune checkpoint molecules, PD-L1 expression has been shown to have both predictive and prognostic value for survival in patients with metastatic GEP-NETs [153]. High PD-L1 expression in gastroentero-pancreatic carcinoma (GEP-NEC) is associated with poor prognosis [154].

The phase 2 KEYNOTE-158 trial assessed the efficacy and safety of pembrolizumab across various cancers, including progressive well-differentiated GEP-NETs. Pembrolizumab alone did not demonstrate a significant benefit in GEP-NETs. Only 3.7% of patients showed a positive response based on the ORR, with no complete responses (CR) and four partial responses (PR)—comprising three cases of pancreatic cancer and one case of rectal cancer—observed after a median follow-up period of 24 months. All patients who responded were PD-L1 negative [155]. The most frequent immune-related adverse events encompassed hypothyroidism, hyperthyroidism, colitis, pneumonia, severe skin reactions, and hepatitis [156]. Moreover, the results from the phase 1b KEYNOTE-028 basket study, which evaluated the safety and efficacy of pembrolizumab in patients with advanced PD-L1-positive (> 1%) NETs, demonstrated that pembrolizumab attained an ORR of 6,3% within the PanNET cohort, with a median response duration of 9.2 months [157].

Furthermore, pembrolizumab, neither in combination with Lenvatinib [158] nor with Lanreotide depot [159], showed a sufficient response in NETs.

Another anti-PD-1 monoclonal antibody, spartalizumab, studied in patients with progressive, well-differentiated metastatic NET G1/2 and GEP-NEC, showed limited activity in the GEP-NET subgroup. The ORR in the NET group was 7.4% (3% in PanNETs), which fell short of the predefined success threshold of ≥10%. The 12-month OS was 73.5% in the NET group [160].

In a phase Ib clinical trial, Toripalimab, which also targets the PD-1 receptor, has demonstrated antitumor activity in the treatment of recurrent or metastatic NENs. A higher ORR was observed in patients exhibiting positive PD-L1 expression, elevated tumor mutational burden (TMB-H), and/or high microsatellite instability (MSI-H) [161].

Due to the limited activity of the monotherapy options mentioned above, combination strategies—such as pairing dual immune checkpoint inhibitors or combining immunotherapy with other therapeutic agents—are being explored to improve the effectiveness of immune checkpoint inhibitors [66], considering their potential for synergy.

A phase II study assessed the effectiveness of combination immunotherapy with anti-cytotoxic T-lymphocyte-associated antigen 4 (CTLA-4) (ipilimumab) and anti-PD-1 (nivolumab) in patients with rare cancers, including advanced NETs, showing an ORR of 43%. The mPFS was 4.8 months, while the median OS was 14.8 months. [162]. The phase 2 DART trial also investigated the dual blockade of CTLA-4 and PD-1 (ipilimumab and nivolumab) in patients with NENs. Reported ORR was 26%, and the 6-month PFS rate was 32% in the high-grade NEN cohort, [163].

An open-label, phase II basket trial involving patients with rare cancers, including PanNETs, evaluated the combination of the immune checkpoint inhibitor atezolizumab and the angiogenesis inhibitor bevacizumab. The combination showed moderate clinical activity in patients with advanced NETs. ORR was observed in 4 (20%) patients with PanNENs. In a safe profile, the mPFS was 14.9 months [164].

Recently, the antibody against PD-L1, avelumab, in combination with the multikinase inhibitor regorafenib, was evaluated in a phase II trial involving advanced GEP-NENs. The results showed a 6-month ORR of 18%, assessed by Response Evaluation Criteria in Solid Tumors (RECIST 1.1) criteria, and the mPFS was 5.5 months. These promising results indicate that combination therapies should be explored further to enhance the efficacy of immune checkpoint inhibitors in PanNETs [165].

Predictive biomarkers for immunotherapy, including PD-L1 expression, MMR status, TMB, as well as aggressive tumor biology, higher T-lymphocyte infiltration, and inflamed tumor microenvironment, are associated with response to immunotherapy [53,166]. In non-small-cell lung cancer, elevated TMB was associated with a benefit from therapy with immune checkpoint inhibitors, independent of PD-L1 expression. Moreover, co-occurrence of TMB and PD-L1 improved the response to immunotherapy [55]. It was shown that high TMB was very rare (≤1.5%) in patients with PanNENs [53] but was higher in patients with G3 NETs and NECs compared to patients with well-differentiated NETs [167]. Most GEP-NETs are characterized as immunologically “cold” tumors, exhibiting a scarcity of tumor-infiltrating lymphocytes and a low TMB, which may contribute to their resistance to immune checkpoint inhibitors [168,169]. Chemotherapy and PRRT are among the therapeutic strategies that may potentially induce an increase in TMB or increase of tumor-infiltrating lymphocytes to enhance the efficacy of immunotherapy [147]. Treatment with immune checkpoint inhibitors may be considered a valuable option for patients with poorly differentiated neoplasms. Meanwhile, for patients with low and intermediate tumor grades, the combination of immunotherapy with other drugs, such as cytotoxic agents, anti-angiogenic therapies, or additional immunotherapy agents, should be explored [170].

Preclinical and clinical studies have demonstrated that temozolomide decreases the number of regulatory T cells and impairs their function [171]. This immunomodulatory effect of temozolomide, along with its effectiveness as a single agent or in combination therapies, indicates potential advantages of combining immune checkpoint inhibitors with this chemotherapeutic agent.

In a non-randomized, phase II study involving patients with NENs, the combination of temozolomide and nivolumab demonstrated promising efficacy, particularly in patients with lung and PanNETs [172]. Because the study has several limitations, including a single-arm design and relatively short follow-up period, further research is needed to confirm that the combination of temozolomide and nivolumab is a valuable therapeutic option for this patient group.

It is suggested that for a selected group of patients with positive PD-L1 expression, TMB, or high MSI, immune checkpoint inhibitors may be an effective treatment option [161].

Treatment with alkylating-based chemotherapy may cause alterations in MMR genes, leading to the development of a hypermutator phenotype. This makes immune checkpoint inhibitors a potentially valuable treatment option for patients with high TMB and MMR changes after treatment with alkylating agents.

A retrospective study assessed patients with advanced PanNETs who had previously received treatment with alkylating agents and had at least one cycle of an immune checkpoint inhibitor. Patients with high TMB, as well as those with altered MMR, exhibited higher ORR compared to patients with low/unknown TMB or no MMR alteration/unknown MMR [173,174].

In an era of advances in innovative medical technologies, new therapeutic options targeting the well-known SSTRs are being developed, offering another potential treatment strategy for NETs. Among these, bispecific antibodies against SSTRs seem to be a promising immunotherapy approach for NETs. Bispecific T-cell engager (BiTE) molecules work by linking an antigen on cancer cells to CD3 on endogenous T cells. This linkage can trigger a strong cytotoxic response from T lymphocytes against cancer cells expressing the antigen [175].

Tidutamab (XmAb18087) is a bispecific antibody targeting SSTR2 and anti-CD3 that stimulates SSTR2-dependent T-cell cytotoxicity, as demonstrated in a monkey model [176], and is currently being tested in patients with NETs in a clinical trial [NCT03411915]. Preliminary results from this study were presented at the 2021 North American Neuroendocrine Tumor Society (NANETS) Annual Symposium and showed that Tidutamab is well tolerated and exhibits moderate antitumor activity [177].

Table 1 summarizes clinical trials investigating immune checkpoint inhibitors for the treatment of neuroendocrine neoplasms.

CAR T-cell therapy represents a new form of cancer immunotherapy currently being investigated in NENs. CARs are recombinant T cells from the patient that are redirected ex vivo to specific antigens on the surface of cancer cells, leading to cytotoxic activity and tumor lysis [178]. CAR T cells targeting SSTRs have demonstrated antitumor activity against human NETs both in vitro and in vivo, but future investigations are needed to confirm their efficacy in patients with PanNETs [179].

Another promising immunotherapeutic approach for NENs is CDH17-dependent CAR T-cell therapy. CDH17-CAR T cells target and destroy PanNET cells expressing CDH17 while sparing normal cells, making this therapy safer [180].

Oncolytic viruses are another innovative immunotherapeutic approach used in cancer treatment. These viruses are engineered to specifically target and infect tumor cells, leading to their rapid destruction [181]. The oncolytic adenovirus AdVince is currently being studied in a phase I/IIa clinical trial involving patients with metastatic NETs (NCT02749331).

## 9. New Targets

Numerous studies are currently exploring treatments for advanced PanNENs. Immunotherapy, new PRRT options, and innovative targeted therapies show promise as research areas. Among these, CDK4/6 inhibitors, DLL3-targeted therapies, and histone deacetylase (HDAC) inhibitors are considered for systemic treatment in this condition.

DLL3, a new therapeutic target, functions as an inhibitory ligand of the Notch pathway [182].

The Notch pathway plays a role in biological processes like cell proliferation and tumor development. Notch activation decreases neuroendocrine differentiation and tumor growth [183]. After binding to different Notch receptors (Notch1-4), DLL3 promotes malignant tumor transformation [49].

Neuroendocrine neoplasms overexpress DLL3, which is linked to tumor progression and generally indicates a poor prognosis, especially in patients with NEC. Targeted therapies against DLL3 seem to be a promising option for NENs [184].

Numerous clinical trials are examining antibody–drug conjugates (ADCs), bispecific T-cell-engaging drugs, trispecific T-cell-engaging drugs, and CAR T-cell therapies targeting DLL3 to assess their effectiveness in patients with NENs.

One of them is Tarlatamab, the first-in-class bispecific T-cell engager that targets DLL3 on tumor cells. After binding to DLL3 on the surface of cancer cells, Tarlatamab activates T-cell lymphocytes, releases inflammatory cytokines, and induces the death of DLL3-expressing cancer cells via cytotoxic T lymphocytes (CTLs) [185]. Tarlatamab showed promising results in a phase I trial [186].

Currently, patients with DLL3-expressing tumors are being enrolled in a multicenter phase II basket trial to assess the safety, tolerability, and effectiveness of Tarlatamab in this group (NCT06788938).

HPN328 is a trispecific T-cell engager targeting DLL3 featuring three binding domains: an anti-DLL3 domain for binding to the target, an anti-CD3 domain for T-cell engagement, and a third domain for albumin to extend its half-life. In an interim analysis, HPN328 showed antitumor activity with an acceptable toxicity profile in patients with advanced cancers expressing DLL3 (NCT04471727) [187].

BI 764532 is another promising agent currently being tested in clinical trials among NET patients who are positive for DLL3 [NCT04429087].

Table 2 summarizes data on DLL3 therapeutic application studies.

The CDK family plays a key role in regulating cell cycle transition, progression, and transcription [44]. Overactivation of CDK4 and CDK6 kinases is associated with a loss of control over cell cycle regulation, contributing to cancer cell growth (140). A clinicopathological study has shown overexpression of CDK4/6 in multiple PanNET cell lines [188]. NETs with an activated CDK4/CDK6-phospho-RB1 pathway may be candidates for cancer therapy with CDK4/CDK6 inhibitors [188]. However, no measurable activity of palbociclib as a single agent has been observed in refractory metastatic PanNETs [189].

Studies show that mutations activating the mTOR pathway may also increase CDK4 expression in PanNET cell lines, indicating a possible synergistic mechanism between CDK4 and mTOR inhibitors [190]. However, this has not been confirmed in clinical trials. A phase II study demonstrated that the combination of ribociclib and everolimus in well-differentiated foregut NETs had moderate activity and was associated with high toxicity [191]. Another CDK4/6 inhibitor, abemaciclib, is currently being evaluated as a monotherapy in patients with advanced, treatment-resistant, and unresectable gastrointestinal NETs in an ongoing phase II trial (NCT03891784). The primary endpoint is ORR.

Mutations in several key genes, including MEN1, DAXX, ATRX, and TP53, which are essential for controlling cell growth, DNA repair, and genome stability, are the primary genetic alterations that cause PanNETs [16]. 

Along with the genetic modifications mentioned above, epigenetic changes that may affect NET pathogenesis include dysregulation of DNA methylation, post-translational modifications of histones, and regulation by non-coding RNAs [19].

Among the enzymes regulating this complex process, histone acetyltransferases (HATs) and HDACs are groups of enzymes that influence epigenetic histone acetylation. HATs add acetyl groups to lysines in histone tails, which leads to DNA relaxation and increased gene transcription [192].

On the other hand, HDACs remove acetyl groups from histones, causing chromatin condensation and leading to gene silencing. HDACs regulate the transcription of genes that encode proteins involved in both tumor initiation and progression [193].

An imbalance in HAT and HDAC activity causes abnormal protein acetylation and is linked to cancer development [194].

Disturbances in HDACs are frequently seen in neoplasms, which is why these enzymes have become a promising target for cancer therapy, and histone deacetylase inhibitors have shown to be effective therapeutic agents in the treatment of cancer [195].

Entinostat, an HDAC inhibitor, was studied in patients with recurrent or refractory abdominal NETs in a phase II trial. Although the trial was terminated prematurely and included only five patients (including five with PanNETs), treatment with entinostat was shown to reduce tumor growth rates and was well tolerated.

Further studies of HDAC inhibitors, especially entinostat, are necessary to confirm their clinical effectiveness in patients with PanNETs [196].

Furthermore, based on the hypothesis that HDAC inhibitors and nicotinamide phosphoribosyltransferase (NAMPT) inhibitors work synergistically in metabolic stress, leading to cell death, the effectiveness of combination therapy was assessed.

Synergistic effects between the two classes of inhibitors have been demonstrated in cell lines derived from neuroendocrine tumors. Therefore, the combination of HDAC and NAMPT inhibitors should be evaluated to develop a new approach to NET treatment [197].

**Figure 1:**
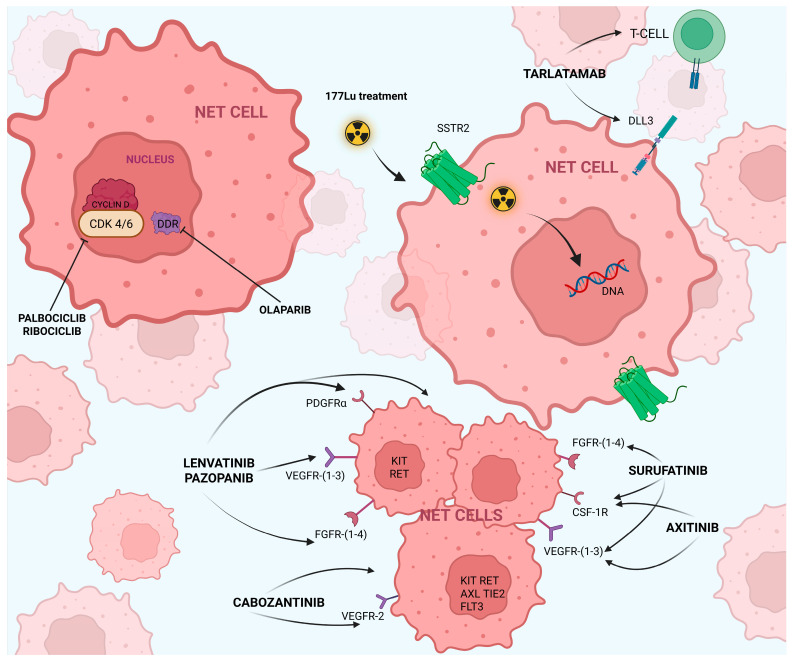
Simplified schematic overview of therapeutic approaches for the management of neuroendocrine tumors (NETs).

Figure 1 summarizes various approaches to treating NETs.

## 10. Perspectives for Clinical and Assistive Implications

The traditional approach to patient therapy, which mainly focused on diagnosing cancer and analyzing specific drivers and biomarkers associated with it, has shifted to a broader approach that considers other patient-dependent factors, such as multimorbidity or non-medical data like lifestyle. In a comprehensive strategy for cancer patients, choosing the right therapy at each stage and monitoring the treatment course—especially regarding side effects—are both very important [198]. The period of the coronavirus pandemic (COVID-19) in 2020 contributed to advances in the development of new digital technologies, including the Internet of Things (IoT) and Artificial Intelligence (AI) [199]. Many e-health technologies are used not only to support healthy behaviors such as promoting physical activity and monitoring diet but also in symptom monitoring for cancer patients [200]. The results of analyses examining the benefits of using IoT in a holistic approach to cancer patient treatment are promising [201,202,203,204]. The latest meta-analysis has reviewed publications examining the impact of IoT use on the survival rates and quality of life improvements for cancer patients undergoing various types of anticancer therapies compared to standard techniques used to assist these patients. Several of the analyzed publications demonstrated a benefit in enhancing patients’ quality of life through IoT by monitoring signs, symptoms, physical activity, and sleep patterns. Since most of these studies are pilot projects, including the devices used for intervention, there remains much to explore to confirm IoT’s role in the comprehensive care of cancer patients [201].

An underestimated aspect of the effectiveness and safety of oncological therapies is ensuring proper nutrition for patients, especially during active treatment, which is also very important for maintaining an adequate quality of life. Although malnutrition affects up to 80% of cancer patients, it is crucial to identify those who need nutritional support through early cachexia detection, risk stratification, and personalized interventions. Among modern technologies, AI—a promising tool in various areas of oncology—has also found its place in this field. AI-based models have been highly accurate in detecting malnutrition, and AI-driven virtual dietitian systems have proven very effective in supporting diet compliance [205]. AI is a field of computer science that uses deep learning and big data analytics, which is becoming increasingly popular in various areas of medicine, including oncology, contributing to solving many biomedical problems [206]. AI tools are being developed to assist clinical decision making and improve its efficiency [207]. It is used at all stages of cancer diagnosis and treatment, from detection and precise molecular characterization of the tumor, through the discovery of new anticancer drugs, to personalizing therapy and predicting patient treatment outcomes [208].

## 11. Conclusions and Future Directions

Numerous systemic therapeutic options are currently available for advanced or metastatic PanNENs; however, the choice of treatment and its sequencing remain unclear.

Despite the availability of multiple systemic treatment methods, there are no clear guidelines for selecting the appropriate therapeutic option at a specific stage of treatment or for the optimal sequence of their use [209]. Furthermore, some differences between the main international NEN guidelines have been highlighted regarding the overall approach, types of options, and the order of their application. The most confusing aspect seems to be choosing the second-line treatment [210]. Therefore, diagnostic and therapeutic decisions should be made within the framework of the multidisciplinary team (MDT). The choice of antiproliferative systemic first-line treatment for patients with PanNETs depends on several features, including tumor grade, Ki-67 index, functionality, growth dynamics, tumor volume, clinical symptoms, and SSTR imaging [211]. The choice of first-line therapy is mainly based on tumor size and how aggressive the tumor is. Due to the effectiveness of SSAs and their favorable toxicity profile, most guidelines recommend SSAs as the first treatment for asymptomatic patients with low proliferative activity (Ki-67 < 10%) and non-bulky disease if they are positive for SSTR expression [212]. Positive SSTR status assessed by SSTR imaging is essential both for targeted treatment with SSAs and for PRRT [213]. Molecularly targeted therapies may be preferred as the first line of treatment for patients with SSTR-negative PanNETs, but they are generally recommended as a second-line therapy. Since both commonly used molecularly targeted agents (everolimus and sunitinib) are approved for the same indication, the choice of a particular drug may depend on its safety profile in the context of comorbidities (including diabetes, metabolic disorders, and hypertension) rather than on disease characteristics [66]. The choice may be even more challenging with the addition of a new molecularly targeted drug: cabozantinib. The history of thromboembolic events and gastrointestinal disorders should be taken into account when considering the potential adverse effects of cabozantinib. For G1/G2 NF-PanNET patients with large, symptomatic tumors and high tumor burden, upfront chemotherapy strategies should be considered, as well as for NEC [214].

Definitive results from ongoing clinical trials are needed to identify the most effective drug combinations and to develop a structured treatment plan tailored to each patient’s characteristics. The use of modern digital technologies can also be helpful in this area.

Various PRRT treatment options are being explored in clinical trials, including next-generation radiopeptides and alpha-emitting agents, which appear to be effective. Monotherapy with checkpoint inhibitors has shown limited antitumor activity, regardless of whether dual blockade of PD-1 and anti-CTLA-4 in patients with NECs can provide therapeutic benefits in advanced NENs. Research into new therapies and drugs, including DLL3-targeted immunotherapy and combining drugs with different mechanisms of action, is essential to improve care for these patients.

Furthermore, optimal treatment plans should be developed by an MDT, considering individual patient characteristics such as performance status or comorbidities and risk-benefit assessments, to create a personalized strategy aimed at achieving long-term disease control and improved clinical outcomes in these patients.

## Figures and Tables

**Table 1:** Clinical trials investigating immune checkpoint inhibitors for the treatment of neuroendocrine neoplasms.

Trial Identifier	Study Phase	Therapeutic Regimen	Type of Therapy or Target	Patient Population	Study Start
NCT05058651	2/3	EP + atezolizumab vs. EP	Chemotherapy + monoclonal antibody for PD-L1	Treatment-naïve, advanced, or metastatic extrapulmonary NEC	28 June 2022
NCT05746208	2	Lenvatinib + Pembrolizumab	Multi-receptor TKI for VEGFR + monoclonal antibody for PD-1	Occurring de novo or great progressive advanced or unresectable WD G3 NET	17 July 2023
NCT05627427	2	Surufatinib + Sintilimab	Small-molecule TKI for VEGFR, FGFR1 + monoclonal antibody for PD-1	Refractory, metastatic, or advanced NET G3, NEC PC	1 July 2022
NCT06889493	2	SVV-001+ Nivolumab + Ipilimumab	Oncolytic virus + monoclonal antibody for PD-1 + monoclonal antibody for CTLA4	Advanced, metastatic, or progressed on at least one line of therapy WD NET G3, NEC	19 May 2025
NCT03591731	2	Nivolumab +/− Ipilimumab	Monoclonal antibody for PD-1+ monoclonal antibody for CTLA4	Refractory, advanced, or metastatic NEC	2 January 2019
NCT06232564	2	Etoposide–carboplatin + Pembrolizumab + Lenvatinib	Chemotherapy + monoclonal antibody for PD-1 + multi-receptor TKI for VEGFR	Metastatic, treatment-naïve for metastatic setting HG-NET	8 July 2024
NCT05015621	3	Surufatinib + Toripalimab vs. FOLFIRI	Small-molecule TKI for VEGFR, FGFR1 + monoclonal antibody for PD-1	Advanced or metastatic, progressed on platinum-based 1st-line chemotherapy NEC	18 September 2021
NCT04525638	2	177Lu-DOTATATE + Nivolumab	PRRT + monoclonal antibody for PD-1	Advanced or metastatic, progressed on at least one line of therapy or treatment-naïve WD NET G3, NEC	29 June 2021
NCT03457948	2	Pembrolizumab + PRRT/arterial embolization/Yttrium-90 microsphere radioembolization	Monoclonal antibody for PD-1+ PRRT + arterial embolization	NET with liver metastases	27 August 2018

Abbreviations: NEC—Neuroendocrine Carcinoma, WD—Well Differentiated, G3—Grade 3, NET—Neuroendocrine Tumor, HG—High Grade, PRRT—Peptide Receptor Radionuclide Therapy, EP—Etoposide + Cisplatin, PC—Pancreatic Carcinoma, PD-L1—Programmed Cell Death Protein-1 ligand, PD-1—Programmed Cell Death Protein-1, CTLA4—Cytotoxic T-lymphocyte-Associated Antigen 4, VEGFR—Vascular Endothelial Growth Factor Receptor, and TKI—Tyrosine Kinase Inhibitor.

**Table 2:** Clinical trials investigating immunotherapy directed to DLL3 in neuroendocrine neoplasms.

Trial Identifier	Study Phase	Therapeutic Regimen	Type of Therapy or Target	Patient Population	Study Start
NCT06788938	2	Tarlatamab	Bispecific T-cell engager	DLL3-Expressing Tumors Including NEN	21 March 2025
NCT06816394	2	Tarlatamab	Bispecific T-cell engager	EPSCC or NEC	15 May 2025
NCT04429087	1	BI 764532	Antibody-like molecule (DLL3/CD3 bispecific)	SCLC and NEN Expressing DLL3	23 September 2020
NCT06132113	1	BI 764532	Antibody-like molecule (DLL3/CD3 bispecific)	NEC	22 January 2024
NCT04471727	1,2	HPN 328	Trispecific T-cell engager	Advanced Cancers Expressing DLL3	14 December 2020
NCT05652686	1,2	Peluntamig	Bispecific antibody	NEC Expressing DLL3	5 September 2023

Abbreviations: DLL3—Delta-like protein 3, NEN—Neuroendocrine Neoplasm, EPSCC—Extrapulmonary Small-Cell Carcinoma, NEC—Neuroendocrine Carcinoma, and SCLC—Small-Cell Lung Carcinoma.

## Data Availability

Not applicable.

## Supplementary Materials

The following supporting information can be downloaded at: https://www.mdpi.com/article/10.3390/cancers17233837/s1, Methods.

## Abbreviations

The following abbreviations are used in this manuscript:

NEN	Neuroendocrine neoplasm
GI-NET	Gastrointestinal neuroendocrine tumor
PanNET	Pancreatic neuroendocrine tumor
GEP-NEN	Gastroentero-pancreatic neuroendocrine neoplasm
GEP-NEC	Gastroentero-pancreatic carcinoma
NET	Neuroendocrine tumor
PanNEC	Pancreatic neuroendocrine carcinoma
WHO	World Health Organization
MEN1	Multiple endocrine neoplasia type 1
VHL	Von Hippel–Lindau syndrome
NF-1	Neurofibromatosis 1
DAXX	Death-domain-associated protein
ATRX	α thalassemia/mental retardation syndrome X-linked
mTOR	Mammalian target of rapamycin
NEC	Neuroendocrine carcinoma
ePanNET	Extrapancreatic neuroendocrine tumor
GEP-NET	Gastroentero-pancreatic neuroendocrine tumor
TAE	Liver-directed transarterial embolization
TACE	Transarterial chemoembolization (TACE)
SIRT	Selective internal radiation therapy
RFA	Radiofrequency ablation
MWA	Microwave ablation
EA	Ethanol ablation
MGMT	O6-methylguanine DNA methyltransferase
CAP-TEM	Capecitabine plus temozolomide
FOLFOX	Fluorouracil plus oxaliplatin
SSA	Somatostatin analog
NF-PanNET	Non-functional pancreatic neuroendocrine tumor
SSTR	Somatostatin receptor
PRRT	Peptide receptor radionuclide therapy
TNM	Tumor, Node, and Metastasis
PFS	Progression-free survival
mPFS	Median progression-free survival
BiTE	Bispecific T-cell engager
NANETS	The North American Neuroendocrine Tumor Society
FDA	Food and Drug Administration
TAT	Targeted alpha therapy
RLT	Radioligand therapy
ENETS	The European Neuroendocrine Tumor Society
ASCO	The American Society of Clinical Oncology
ESMO	The European Society of Medical Oncology
PI3K	Phosphoinositide 3-kinase
VEGF	Vascular endothelial growth factor
VEGFR	Vascular endothelial growth factor receptor
PDGF	Platelet-derived growth factor
PDGFR	Platelet-derived growth factor receptor
FGF	Fibroblast growth factor
FGFR1	Fibroblast growth factor receptor 1
TKI	Tyrosine kinase inhibitor
c-kit	Stem cell factor receptor
ORR	Objective response rate
c-MET	Mesenchymal–epithelial transition
CSF-1R	Colony-stimulating factor 1 receptor
OS	Overall survival
RCC	Renal cell carcinoma
HIF	Hypoxia-inducible factor
PPGL	Pheochromocytoma/paraganglioma
wt-GIST	Wild-type gastrointestinal stromal tumor
CAR	Chimeric antigen receptor
CTLA-4	Cytotoxic T-lymphocyte-associated antigen 4
PD-1	Programmed cell death protein -1
PD-L1	Programmed cell death protein -1 ligand
CR	Complete response
PR	Partial response
TMB	Tumor mutational burden
TMB-H	High tumor mutational burden
MSI	Microsatellite instability
RECIST	Response Evaluation Criteria in Solid Tumors
MMR	Mismatch repair
CDK	Cyclin-dependent kinase
DLL3	Delta-like ligand 3
HDAC	Histone deacetylase
ADC	Antibody–drug conjugate
CTL	Cytotoxic T lymphocyte
HAT	Histone acetyltransferases
NAMPT	Nicotinamide phosphoribosyltransferase
IoT	Internet of Things
AI	Artificial intelligence
MDT	Multidisciplinary team
