# Supplementary material for: New Treatment Options for Pancreatic Neuroendocrine Tumors: A Narrative Review
Source: Cancers (Basel). 2025 Nov 29;17(23):3837. doi: 10.3390/cancers17233837 (PMC12691000; doi:10.3390/cancers17233837)
Supplement: Supplementary file 1 [file cancers-17-03837-s001.zip › cancers-3961352-supplementary.pdf]

Methods

This review was conducted using a narrative approach to summarize and contextualize current evidence on systemic treatment options for PanNET. While it was not carried out as a formal systematic review, a structured and transparent search strategy was used to ensure the inclusion of the most relevant and recent literature.

Literature Search Strategy

The literature was gathered through a targeted search of PubMed, Scopus, and Google Scholar from their inception until July 2025. The search focused on studies related to systemic therapies for PanNET, including randomized clinical trials, meta-analyses, and significant observational studies. Search terms included combinations like "metastatic PanNET," "pancreatic neuroendocrine tumors," "systemic treatment options," "targeted therapies," "somatostatin analogs," "tyrosine kinase inhibitors," "mTOR inhibitors," and "clinical trials." Only articles published in English were included. To identify the latest and ongoing therapeutic developments, ClinicalTrials.gov was also checked for currently registered and recently completed clinical trials.

Study Selection Approach

Within the narrative framework, the selection of studies was conducted manually by two authors, who independently reviewed titles and abstracts to identify articles on systemic treatment strategies. The full texts of potentially eligible articles were then assessed. Studies were included if they mainly focused on PanNET and described or evaluated therapeutic approaches, including targeted therapies, somatostatin analogs, PRRT, chemotherapy, immunotherapy, or emerging agents. Articles were excluded if they were non-English, discussed non-systemic or purely surgical interventions, or were not relevant to current therapeutic strategies.

Data Extraction and Synthesis

Data extraction followed a qualitative approach. Key findings from each included study, such as treatment efficacy, safety data, mechanisms of action, and clinical applicability, were summarized descriptively. The evidence was then thematically synthesized to highlight established treatments, evolving therapeutic strategies, and ongoing trends in clinical research.
